# Comparison of the accuracy of bracket positioning between direct and digital indirect bonding techniques in the maxillary arch: a three-dimensional study

**DOI:** 10.1186/s40510-022-00426-3

**Published:** 2022-09-05

**Authors:** Rami Aboujaoude, Roland Kmeid, Carine Gebrael, Elie Amm

**Affiliations:** 1 Department of Orthodontics and Dento-facial Orthopedics, Saint Joseph University of Beirut Faculty of Dental Medicine, Beirut, Lebanon; 2 Department of Orthodontics and Dento-facial Orthopedics, Boston University Henry M. Goldman School of Dental Medicine, Boston, USA

**Keywords:** Digital bonding, Direct bonding, 3Shape, OrthoAnalyzer, Exocad

## Abstract

**Background/objectives:**

When the indirect bonding technique was developed in 1972 by Silverman and Cohen, many authors wondered whether this technique would improve bracket positioning accuracy compared to the direct bonding technique. Studies have found little to no difference between them regarding positioning accuracy. Recently, technological advances have improved the indirect method by allowing the user to position the brackets virtually using software applications such as OrthoAnalyzer™. To the best of our knowledge, no studies have compared direct positioning to this new digital indirect technique. Thus, the aim of this study was to compare the accuracy of placement between the two techniques in the maxillary arch using two different bracket types: conventional twin brackets and self-ligating brackets. A secondary objective was to evaluate whether bracket type affected positioning accuracy.

**Methods:**

A maxillary arch of a patient was scanned by digital impression. Forty resin duplicates of this model were printed and then mounted on a mannequin head, on which 20 practitioners performed direct bonding using the aforementioned brackets. Later on, they performed a virtual indirect bonding of the same case virtually, with the digital impression superimposed to the patient’s CBCT (cone-beam computed tomography). Afterwards, the direct bonded models were unmounted, scanned, and then superimposed to the indirect models. Differences in height, angulation and mesio-distal position of the brackets were evaluated.

**Results:**

Regarding height, the differences between direct and indirect methods were not significant. Height difference was significantly greater for self-ligating brackets compared to conventional brackets. Regarding mesio-distal positioning, significant differences were noted for teeth 13 and 15 with self-ligating brackets (*p*-value = 0.019 and *p*-value = 0.043, respectively). The deviation was also greater for these brackets. Regarding angulation, the difference was significant on tooth 12 when using conventional brackets (*p*-value = 0.04) and on 12 and 22 when using self-ligating brackets (*p*-value = 0.09).

**Conclusion/implications:**

There were no major significant differences between direct and indirect bonding. Differences were significant only on the laterals for of angulation, and on teeth 13 and 15 for mesio-distal centering. The bracket type seems to influence positioning accuracy, since self-ligating brackets had a larger deviation range than conventional brackets.

## Background

The science of orthodontics is not a recent discipline. The earliest records of moving teeth go back to the year 25 BC where people were told to apply constant pressure with their finger on crowded teeth for better alignment [1].

In the nineteenth century, a number of removable appliances emerged on the market, which were considered groundbreaking at the time, but allowed for very poor root control [2]. This is when Edward Angle’s edgewise appliance truly revolutionized orthodontics, allowing the orthodontist to accurately control teeth in all three dimensions, by incorporating bends in the archwire [3]. Despite being revolutionary, this technique was deemed by some to be time-consuming and demanding of manual dexterity. Thus, the straight wire technique was developed by Lawrence Andrews in the 70s with the advantage of reducing the overall number of bends [4].

After being studied extensively by Andrews and many others, it was shown that the straight wire technique depended on accurate bracket placement [3]. A study by Meyer and Nelson showed that a vertical positioning error of 3 mm on a premolar led to a 15 degree torque variation and a 0.04 mm first-order variation [5]. The transition from banding to bonding in orthodontics pushed researchers toward improving bonding quality, in order to insure adequate bracket placement, which became a fundamental issue in orthodontics, as TM Graber puts it: “… the best results in the present and in the future will be achieved by those orthodontists who are best at accurate bracket positioning” [6, 7].

In this context, the indirect bonding technique was developed by Silverman in 1972. Among many of its claimed advantages, precise bracket placement stood out, given that the positioning was being done extra-orally [8]. Despite this claim, many studies failed to show major differences between direct and traditional indirect bracket positioning techniques: A study by Aguirre et al. found that neither technique achieved ideal bracket placement. An in vitro study by Koo et al. found no differences between the two techniques regarding angulation and mesio-distal positioning. A randomized clinical trial by Hodge et al. also did not report significant differences between the two techniques [9–12].

However, it should be stated that in these studies, the traditional indirect bonding protocol was followed, which had the drawback of possible unwarranted imprecisions, since brackets were placed manually on the cast. Furthermore, no radiological support was provided during bracket positioning. Studies have shown that even a panoramic X-ray may not be an accurate guide for bracket placement [13]. Finally, it should be kept in mind that all these studies used only one bracket type in their protocol; a comparison between direct and indirect techniques using different brackets would be interesting since it has been shown that bracket type may influence positioning accuracy [14].

With the introduction of cone-beam computed tomography (CBCT) and the rise of digital dentistry, technological advances have pushed the limits of indirect bonding even farther by allowing the orthodontist to place brackets virtually, with a three-dimensional visual access to the roots using software applications such as OrthoAnalyzer™ [15]. To the best of our knowledge, no studies have compared direct bracket positioning to the new aforementioned digital indirect technique.

Therefore, the main objective of this clinical in vitro study is to compare the accuracy of placement of two different bracket types, conventional and self-ligating, in the maxillary arch, between direct and digital indirect positioning techniques, using a 3D software. The secondary objective is to evaluate whether bracket type affects positioning accuracy.

## Materials and methods

This study was conducted in the Department of Orthodontics and Dentofacial Orthopedics at the Saint-Joseph University of Beirut, Lebanon. Ethical approval was obtained from the Saint-Joseph Ethics Committee (Approval No. 2020-150).

The first step was to select a patient’s pretreatment model. The following inclusion criteria were taken into consideration:

- Presence of all the upper teeth from right second premolar to left second premolar;
- Presence of an ethically justified CBCT (cone-beam computed tomography) examination.

However, the exclusion criteria were as follows:

- Severe crowding which would render the bonding difficult;
- Teeth with bizarre anatomy or severely decayed;
- History of orthodontic treatment.

After searching in the data of the orthodontic department at the Saint-Joseph University of Beirut, the following patient met the above criteria and was thus chosen for the study:

H.H. (27 years old) presented to the orthodontic department complaining of his narrow smile. He was diagnosed as having a skeletal crossbite. The treatment plan included a mini-screw-assisted rapid palatal expansion (MARPE).

Studies have shown that the use of a CBCT examination would be helpful in designing a digital guide for the insertion of the mini-screws in the palate [16]. Thus, an ethically justified CBCT was obtained.

**Fig. 1:**
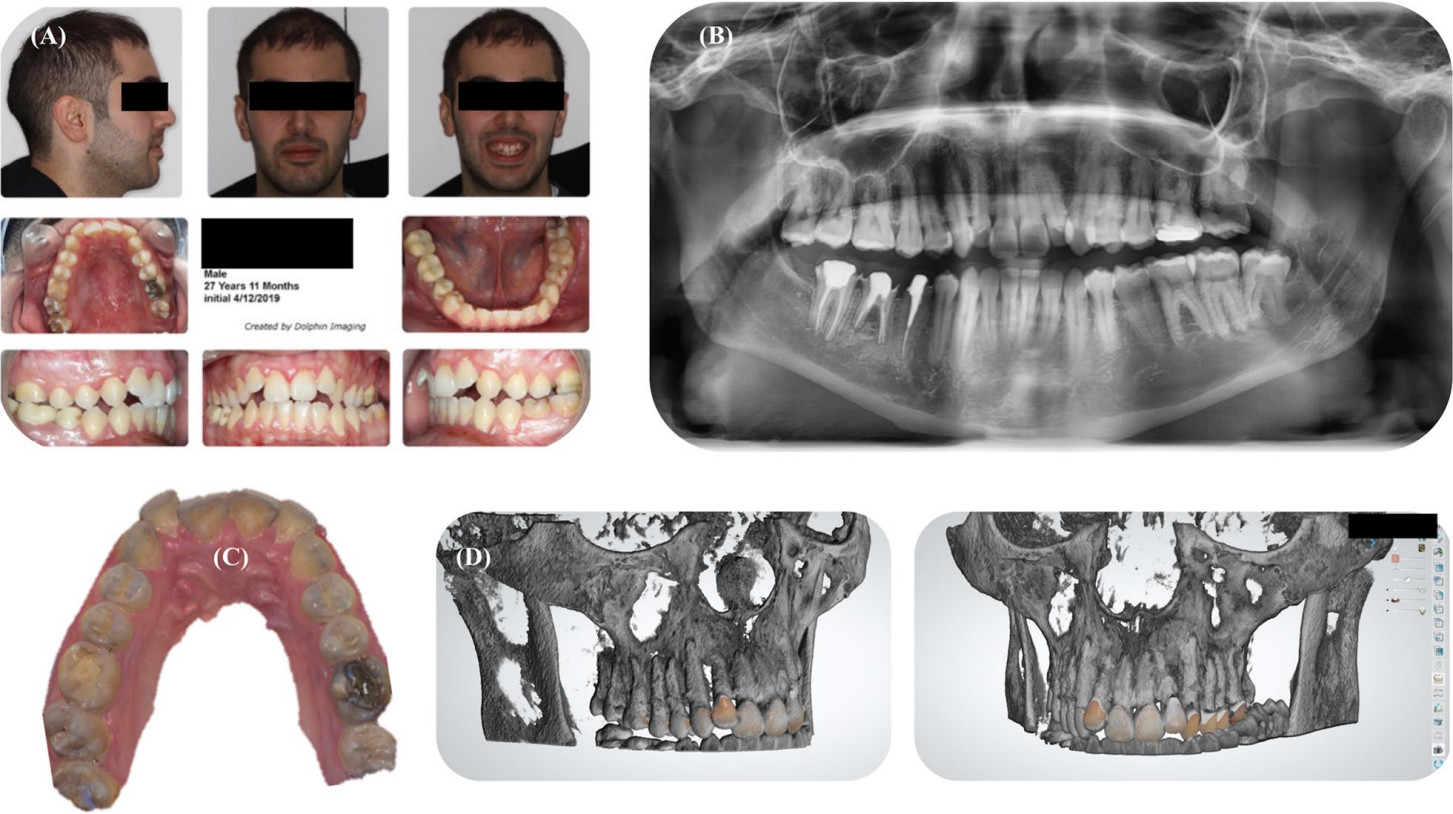
Pretreatment data of the patient. **A** Intra-oral and extra-oral photographs of the patient. **B** Panoramic radiograph. **C** Maxillary intra-oral scan. **D** Superimposition of the CBCT and the impression using OrthoAnalyzer ™ software

A digital impression of the patient’s maxillary arch was taken, and the CBCT was superimposed to it using the OrthoAnalyzer ™ (3Shape; Copenhagen, Denmark) software (Fig. 1).

The second step was to choose the types of brackets used in this study. The selection was based on the availability of the brackets in both the OrthoAnalyzer ™ library and the Lebanese market and on the popularity of these bracket types among Lebanese orthodontists.

A questionnaire was sent out to 30 orthodontists, in order to determine the types of brackets most commonly used in their practice. The results showed that the conventional metallic twin brackets and the self-ligating metallic brackets were the most popular. Therefore, conventional metallic brackets “Mini-Master 0.022 MBT (McLaughlin Bennett Trevisi prescription) Compatible” (American Orthodontics Corporation, Sheboygan, WI, USA) and self-ligating metallic brackets “Damon Q2 0.022 Standard Torque” (Ormco Corporation, Orange, CA, USA) were chosen.

The third step was to choose the orthodontists who would be included in the study.

The inclusion criteria were the following:

- Specialist orthodontist;
- Having a minimum of 2 years of clinical experience;
- Having already used the OrthoAnalyzer™ software;
- Having already used the two types of brackets chosen in the study.

A total of 20 orthodontists were chosen.

This study was divided into two phases: the direct bonding phase first, followed by the indirect bonding phase.

*Direct bonding*. 40 duplicate resin models were printed (*Formlabs 2 3D printer*) and mounted on a mannequin head (*Frasaco P-6/3 PT Premium Head with Bench Assembly*).

The practitioners, after having signed an informed consent form, were asked to position the brackets, using a gauge (*Swivel Head Bracket Gauge, Ortho Organizer*) according to the following heights: 4 mm for the central incisors and the premolars, 3.5 mm for the lateral incisors and 4.5 mm for the canines. They were asked to position the brackets in the mesio-distal center of each tooth, and with the angulation they judge to be correct based on the panoramic X-ray of the patient.

Composite (*Quick-Cure, Reliance Orthodontic Products*) was used.

**Fig. 2:**
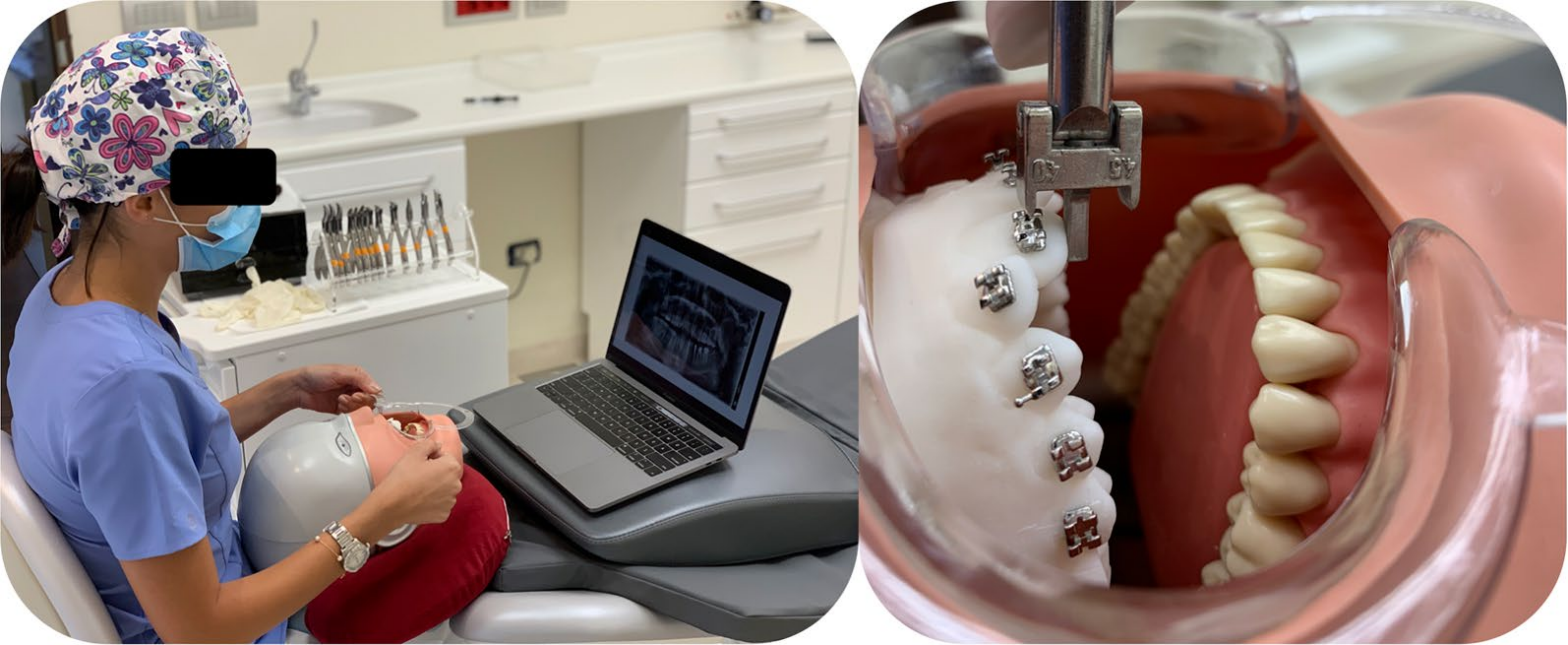
Direct bonding performed on a mannequin head

The orthodontist bonded the whole arch (from tooth 15 to tooth 25) twice, using both bracket types; thus, a total of two direct bondings per practitioner (Fig. 2).

**Fig. 3:**
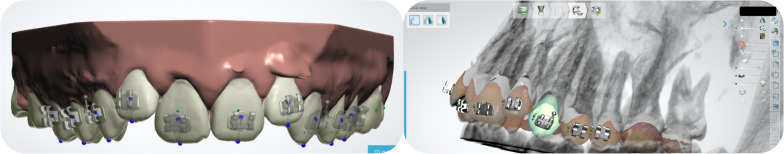
Indirect digital bonding performed on OrthoAnalyzer™

*Indirect bonding.* Two weeks later, after segmentation of the digital impression and superimposition of the CBCT, the participants were calibrated on using the software by a technician, who remained by their side during bracket placement in case of any technical question. The aforementioned brackets were chosen from the library of the OrthoAnalyzer™ software, then positioned by the 20 orthodontists based on the same height measurements, at the mesio-distal center of each tooth, and according to the 3D angulation of the root. The orthodontist bonded the whole arch (from tooth 15 to tooth 25) twice, using both bracket types, thus a total of 2 indirect bondings per practitioner (Fig. 3).

**Fig. 4:**
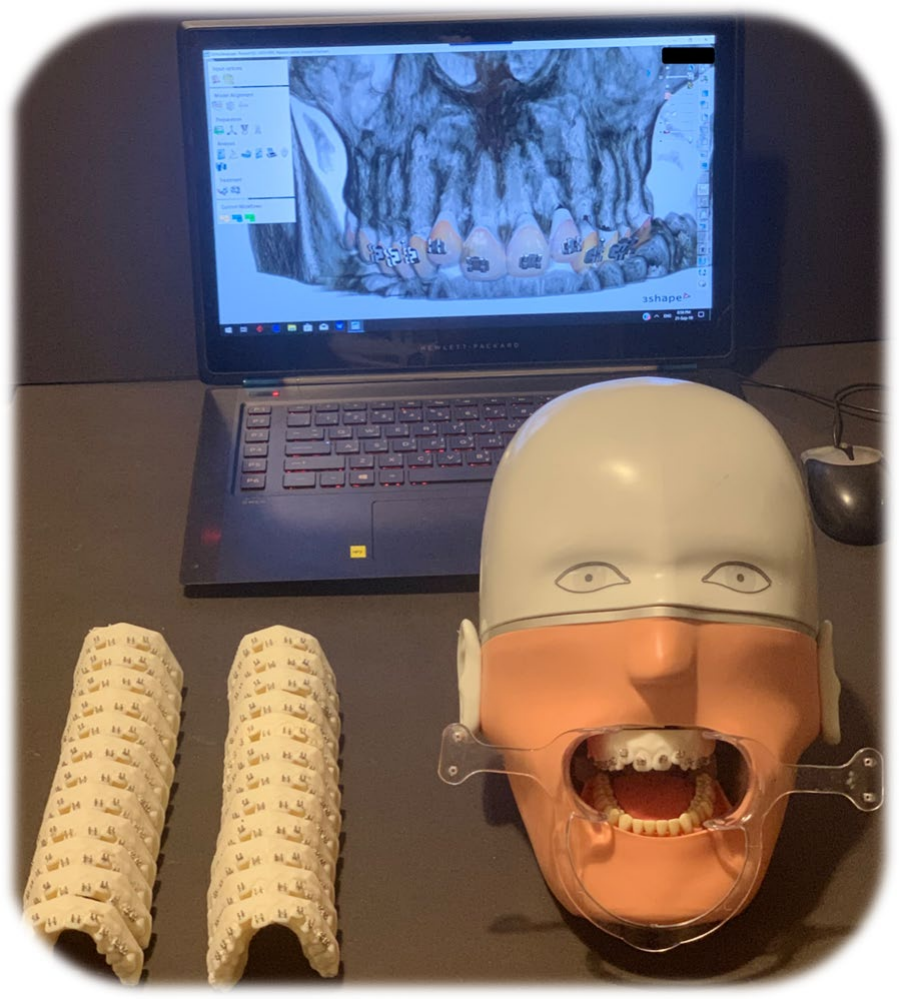
Unmounted direct bonded models that were scanned by a Trios digital scanner

**Fig. 5:**
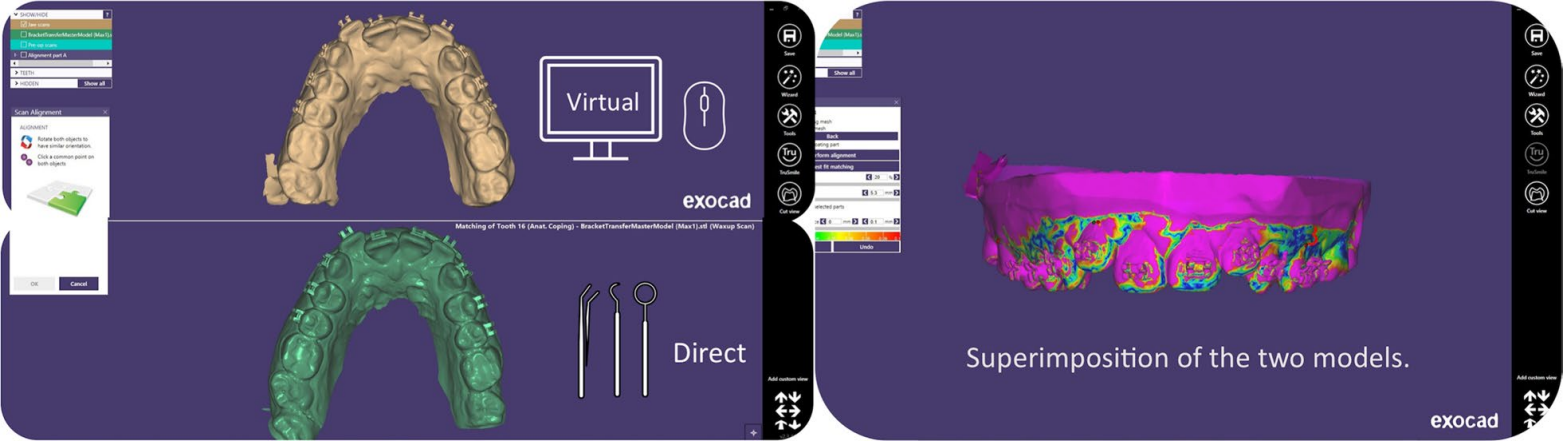
Superimposition of the models on the Exocad*™* software

Afterwards, the direct bonded models were unmounted from the mannequin head and scanned by a Trios digital scanner (*3Shape; Copenhagen, Denmark*) (Fig. 4). These models were then superimposed to the virtual indirect bonded models, in order to assess the differences using the Exocad™ software via the “best fit matching” option (Fig. 5).

**Fig. 6:**
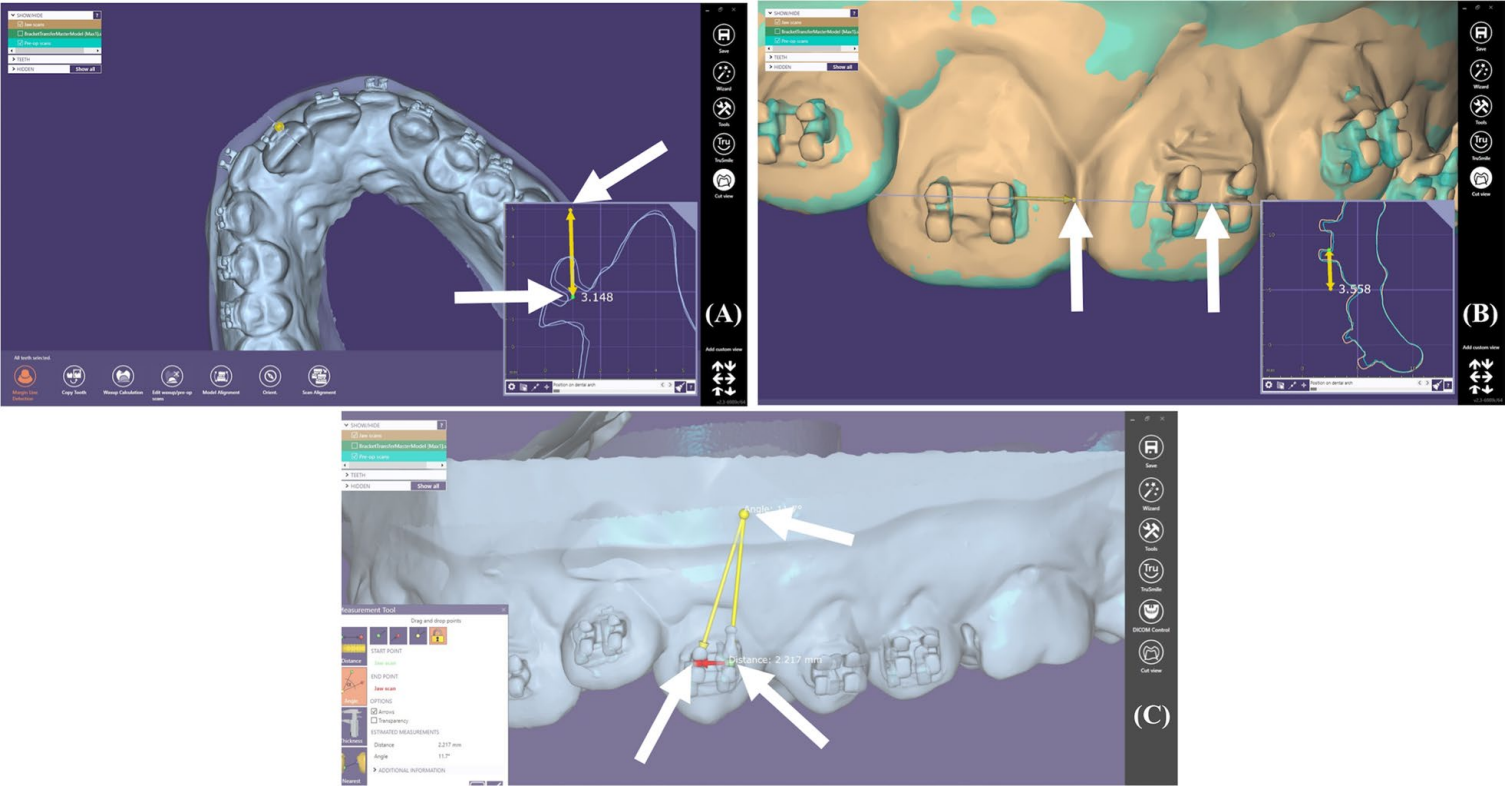
Different measurements. **A** Height. **B** Mesio-distal positioning. **C** Angulation

Two linear and one angular differentials between the direct and indirect superimposition were measured by an operator (*RA*):

- Height: A vertical cross section was made vertical through the center of the brackets; two points were chosen: the first on the incisal edge, or cusp, and the second on the center of the bracket.
- Mesio-distal positioning: A horizontal cross section was made through the center of the brackets; two points were chosen: the first on the mesial contact point, and the second on the center of the bracket.
- Angulation: the angulation option was used for measurements, placing two points on the centers of the mesial and distal edges of the brackets and a fixed point on the 3D root (Fig. 6).

All of the above measurements were noted; then, 10 models were chosen randomly and all measures were re-evaluated by a second practitioner (CG) in order to assess the interclass correlation and guarantee accurate measurements.

### Statistical analysis

A power posttest was calculated for this analysis and was superior to 80%. The study was designed to have a permissible risk of error α of 5%. SPSS® version 27.0 software was used to statistically analyze the data. Accepted limits for the grouping of ranges included a deviation of 0.5 mm for the linear dimensions and 2° for angulation, as per the ABO (American Board of Orthodontics) recommendations [17].

The following tests were applied:

- Kolmogorov–Smirnov test to assess the normality of distribution of the quantitative variables.
- Repeated measures analysis of variance with two within-subjects’ factors.
- One-sample t tests were carried out in order to compare the measurement deviations of the height and the mesio-distal centering with the theoretical value of “0.5 mm.” Also, one-sample t tests were applied in order to compare the angulation differences with the theoretical value of "2°".
- Interclass coefficient with 95% confidence interval to assess the measurements reproducibility.

## Results

The interclass coefficient values are shown in Table 1. A good reproducibility was noted for the three parameters and for the two types of brackets, conventional brackets (MBT) and self-ligating brackets (Damon).

**Table 1:** Interclass coefficient values for each bracket type and for each parameter

Bracket	Parameter		
	Height	Angulation	Mesio-distal positioning
MBT	0.960 (0.936–0.975)	0.975 (0.960–0.985)	0.729 (0.564–0.832)
Damon	0.921 (0.873–0.951)	0.984 (0.974–0.990)	0.951 (0.922–0.970)

### Comparison of the height deviation

The mean and standard deviation of the difference in height between the direct method and the indirect method using the MBT or Damon brackets placed on the maxillary teeth are shown in Table 2.

**Table 2:** Mean and standard deviation of the differences in height between direct and indirect methods using MBT and Damon brackets

Tooth	Height deviation (mm)	N	Mean	Sd	Comparison between MBT and Damon brackets	Comparison with the theoretical value 0.5 mm
					*p*-value	*p*-value
15	MBT	20	0.433	0.183	0.112	0.117
	DAMON	20	0.501	0.142		0.975
14	MBT	20	0.382	0.141	0.003	0.001
	DAMON	20	0.502	0.148		0.964
13	MBT	20	0.187	0.110	0.039	< 0.001
	DAMON	20	0.240	0.074		< 0.001
12	MBT	20	0.234	0.151	0.054	< 0.001
	DAMON	20	0.296	0.089		< 0.001
11	MBT	20	0.141	0.088	0.023	< 0.001
	DAMON	20	0.189	0.081		< 0.001
21	MBT	20	0.179	0.147	0.486	< 0.001
	DAMON	20	0.204	0.110		< 0.001
22	MBT	20	0.163	0.082	0.000	< 0.001
	DAMON	20	0.260	0.110		< 0.001
23	MBT	20	0.261	0.149	0.168	< 0.001
	DAMON	20	0.302	0.167		< 0.001
24	MBT	20	0.337	0.198	0.003	0.002
	DAMON	20	0.486	0.165		0.699
25	MBT	20	0.440	0.223	0.010	0.239
	DAMON	20	0.488	0.222		0.812

**Fig. 7:**
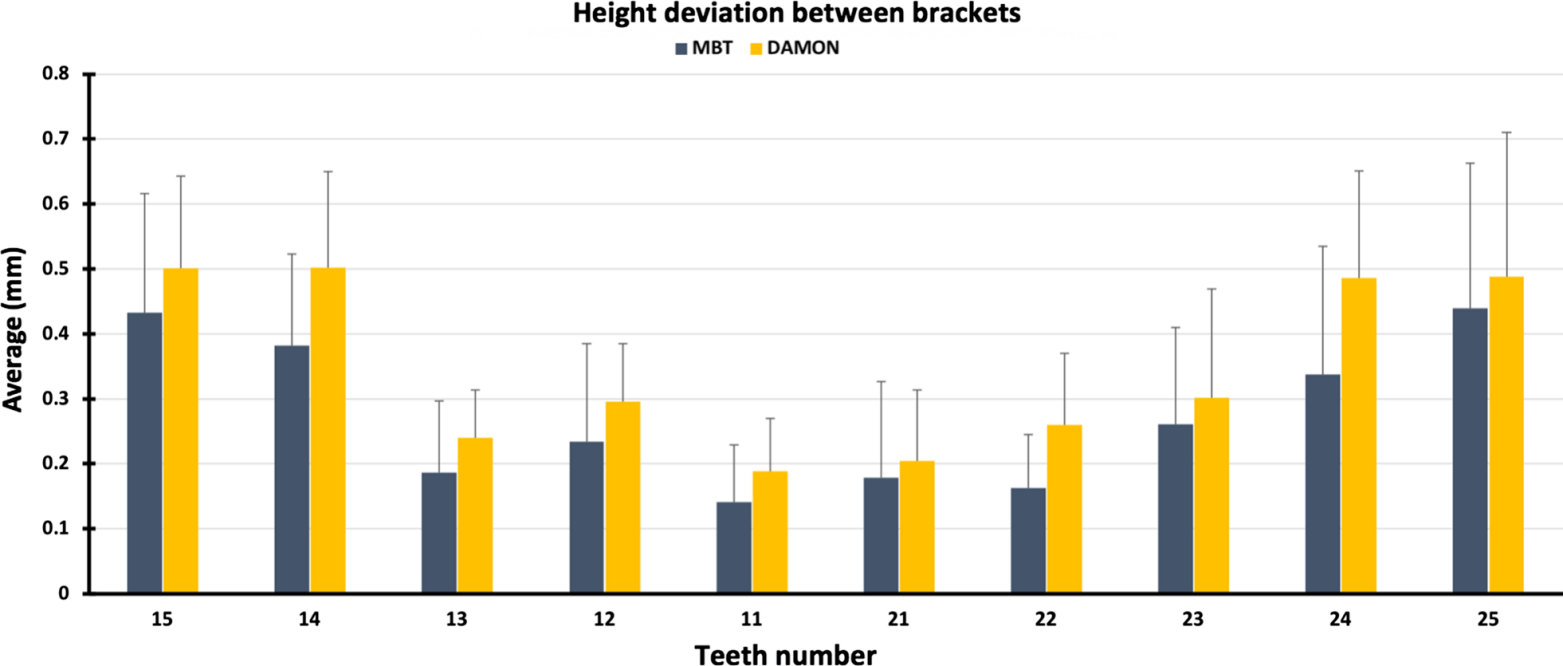
Differences in height on each tooth between direct and indirect methods using MBT and Damon brackets

The one-sample t tests showed that the height difference between the direct method and the indirect method was not significantly greater than the theoretical value of “0.5 mm” for all of the teeth and for both brackets. The results showed that the height difference was significantly greater for the Damon brackets compared to the MBT brackets on the following teeth: 11 (*p*-value = 0.023), 13 (*p*-value = 0.039), 14 (*p*-value = 0.003), 22 (*p*-value < 0.001), 24 (*p*-value = 0.003) and 25 (*p*-value = 0.010) (Fig. 7).

### Comparison of the mesio-distal positioning deviation

The mean and standard deviation of the difference in mesio-distal positioning between the direct method and the indirect method when using MBT or Damon brackets placed on the maxillary teeth are presented in Table 3.

**Table 3:** Mean and standard deviation of the differences in mesio-distal positioning between direct and indirect methods using MBT and Damon brackets

Tooth	Mesio-distal positioning deviation	N	Mean	Sd	Comparison between MBT and Damon brackets	Comparison with the theoretical value 0.5 mm
					*p*-value	*p*-value
15	MBT	20	0.320	0.149	< 0.001	< 0.001
	DAMON	20	0.565	0.133		0.043
14	MBT	20	0.304	0.150	0.001	< 0.001
	DAMON	20	0.508	0.142		0.815
13	MBT	20	0.373	0.116	< 0.001	< 0.001
	DAMON	20	0.560	0.104		0.019
12	MBT	20	0.234	0.097	< 0.001	< 0.001
	DAMON	20	0.508	0.105		0.754
11	MBT	20	0.277	0.072	< 0.001	< 0.001
	DAMON	20	0.536	0.117		0.184
21	MBT	20	0.312	0.143	< 0.001	< 0.001
	DAMON	20	0.501	0.083		0.957
22	MBT	20	0.371	0.129	< 0.001	< 0.001
	DAMON	20	0.531	0.093		0.151
23	MBT	20	0.384	0.118	0.005	< 0.001
	DAMON	20	0.492	0.106		0.740
24	MBT	20	0.352	0.139	0.002	< 0.001
	DAMON	20	0.499	0.109		0.952
25	MBT	20	0.332	0.176	< 0.001	< 0.001
	DAMON	20	0.525	0.079		0.173

The one-sample t tests showed that the deviation of the mesio-distal positioning between the direct method and the indirect method was not significantly different from the theoretical value “0.5 mm” with the Damon brackets placed on all the teeth except for teeth 13 (*p*-value = 0.019) and 15 (*p*-value = 0.043).

**Fig. 8:**
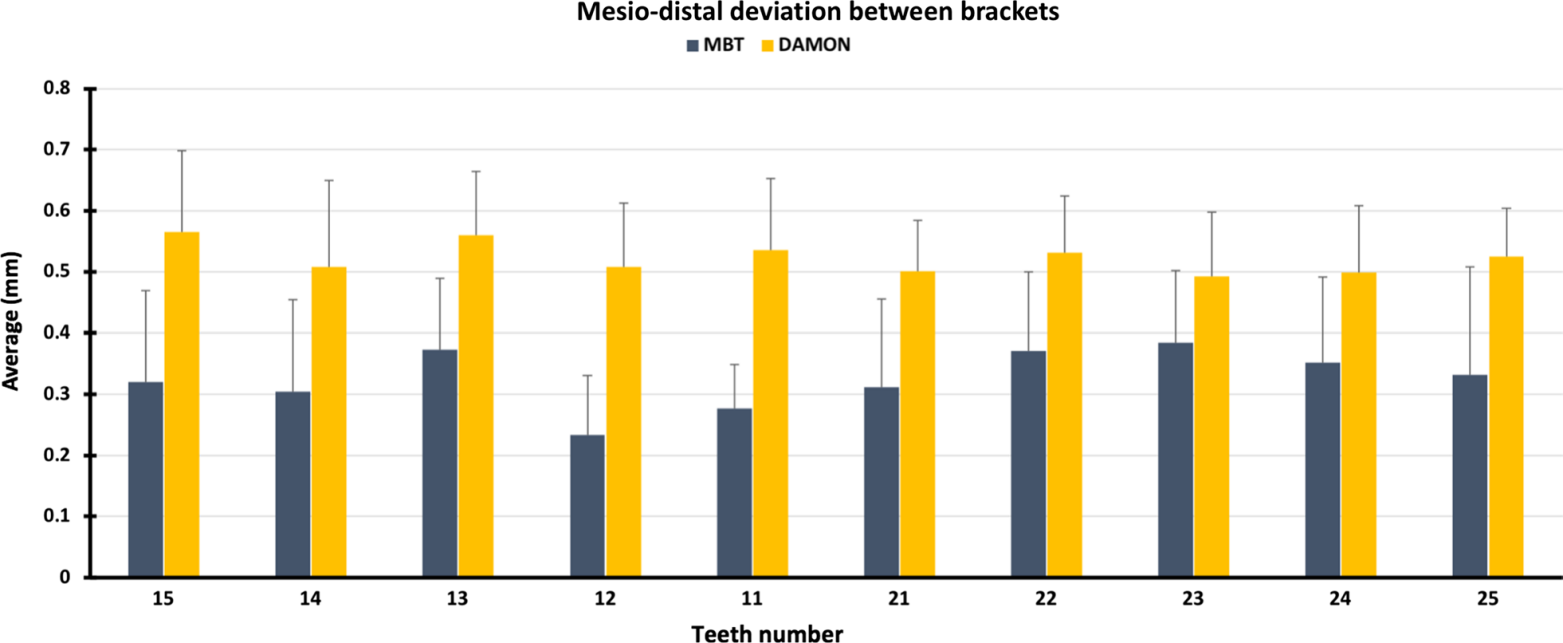
Differences in mesio-distal positioning on each tooth between direct and indirect methods using MBT and Damon brackets

The deviation of the mesio-distal centering was significantly greater for Damon brackets compared to the MBT brackets for the following teeth: 11 (*p*-value < 0.001),12 (*p*-value < 0.001), 13 (*p*-value < 0.001), 14 (*p*-value = 0.001), 15 (*p*-value < 0.001), 21 (*p*-value < 0.001), 22 (*p*-value < 0.001), 23 (*p*-value = 0.005), 24 (*p*-value = 0.002) and 25 (*p*-value < 0.001) (Fig. 8).

### Comparison of the angulation deviation

The mean and standard deviation in the angulation deviation between the direct method and the indirect method of MBT and Damon brackets placed on the maxillary teeth are described in Table 4.

**Table 4:** Mean and standard deviation of the differences in angulation between direct and indirect methods using MBT and Damon brackets

Tooth	Angulation deviation	N	Mean	Sd	Comparison between MBT and Damon brackets	Comparison with the theoretical value 2 degrees
					*p*-value	*p*-value
15	MBT	20	1.572	0.260	0.655	< 0.001
	DAMON	20	1.532	0.324		< 0.001
14	MBT	20	1.517	0.283	0.287	< 0.001
	DAMON	20	1.636	0.420		0.001
13	MBT	20	1.936	0.357	0.121	0.433
	DAMON	20	1.732	0.481		0.022
12	MBT	20	2.780	0.517	0.004	< 0.001
	DAMON	20	2.347	0.507		0.006
11	MBT	20	1.583	0.269	0.752	< 0.001
	DAMON	20	1.611	0.298		< 0.001
21	MBT	20	1.478	0.282	0.710	< 0.001
	DAMON	20	1.438	0.309		< 0.001
22	MBT	20	1.910	0.536	0.009	0.460
	DAMON	20	2.388	0.711		0.025
23	MBT	20	1.586	0.380	0.386	< 0.001
	DAMON	20	1.485	0.311		< 0.001
24	MBT	20	1.533	0.389	0.693	< 0.001
	DAMON	20	1.502	0.286		< 0.001
25	MBT	20	1.324	0.581	0.361	< 0.001
	DAMON	20	1.418	0.308		< 0.001

The one-sample t tests showed that the difference in angulation between the direct method and the indirect method was significantly smaller than “two degrees” with the two types of brackets on all the teeth excluding tooth 12 when using MBT brackets, and teeth 12 and 22 when using Damon brackets.

**Fig. 9:**
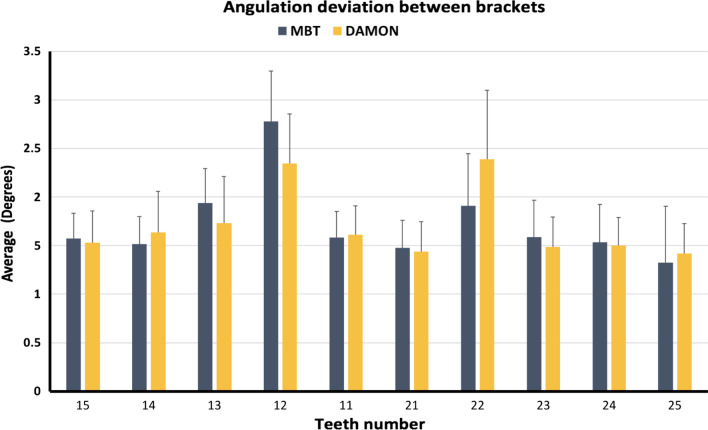
Differences in angulation on each tooth between direct and indirect methods using MBT and Damon brackets

The results showed that the angulation difference was not significantly different between the Damon and MBT brackets on all the teeth except for the laterals where the difference was significantly larger with MBT brackets compared to Damon brackets (Fig. 9).

## Discussion

The straight wire technique was designed to reduce the number of bends required in the archwire by incorporating information directly into the brackets [18]. However, the results of this treatment technique depend on several factors, notably the accurate positioning of brackets [3].

For Gianelly, the main advantage of the indirect technique would be better bracket positioning accuracy [19]. However, few studies have been able to validate this [9–12].

Recent technological advances have pushed the limits of indirect bonding even farther by allowing the orthodontist to do a virtual bracket placement with a visual 3D access to the roots using software applications such as OrthoAnalyzer™ [15]. To the best of our knowledge, no studies compared direct bracket positioning to the new aforementioned digital indirect technique.

The aim of this study was to assess whether there are any statistically significant differences between direct and digital indirect placement techniques of two bracket types. Three parameters were studied: height, angulation and mesio-distal positioning of the brackets. The influence of the bracket type on the positioning accuracy was also evaluated as a secondary objective.

In this study, the “best fit matching” option in the Exocad™ software was used for the superimpositions: The user is asked to place as many reference points as needed on the two models. The software then performs an accurate and precise alignment. Other software applications, such as the OrthoAnalyzer™ software, only allow the user to place a total of three alignment points, which may hinder accurate superimposition.

Measurements were taken on 2D cross sections rather than using the 3D option. This was done because we noticed a larger inter-operator variation using the 3D option, since the distance was evaluated between two points that can be moved in all three dimensions, whereas the 2D cross sections allowed for precise and reproductible measurements that were automatically calculated by the software.

In terms of height, the difference between the direct and indirect method was less than, or equal to 0.5 mm for both Damon and MBT brackets on all of the maxillary teeth. This means that there are no statistically significant differences in height between the two techniques, which is consistent with the results obtained by Hodge et al. and Aguirre et al. [11, 12] but not consistent with those reported by Koo et al. [10]. A possible explanation for that could be the fact that Boone gauges were used in the latter study, and a study by Mohammadi et al. found that height positioning gauges were more accurate than Boone gauges [20]. In the present study, the “distance from incisal edge” option was used in the OrthoAnalyzer™ software, through which the user enters the desired bracket height, and the software automatically positions the brackets accordingly. Since no differences were found between direct and indirect techniques, this could be additional validation that height positioning gauges, which were used in this study, are a precise tool for positioning brackets in the vertical dimension. This was also reported by Armstrong et al. [21].

Regarding mesio-distal positioning, the difference between the two methods was significant on teeth 13 and 15. This was not reported in the literature, but it should be said that some studies did not consider horizontal bracket positioning mistakes due to the difficulty of measuring said parameter with overlapping teeth [11, 22]. Koo et al. resorted to manually sectioning the teeth using a saw, which was a strenuous process [10]. The use of technology in our study enabled us to virtually segment the teeth, with ease and precision, using software applications. This may explain why our results are not concurrent with those found in the literature. Nevertheless, Aguirre reported that in both direct and indirect technique, linear measurements of bracket positioning tended to be more precise on the left side than that on the right side for the maxillary arch [11]. Furthermore, Dr. Tom Pitts reported that placing brackets too mesially on the premolars and too distally on the canines were common mistakes made by orthodontists during direct bonding [23].

In terms of angulation, the difference between the two methods was significant for MBT brackets on the upper right lateral, and for Damon brackets on both upper laterals. The results in the literature showed no difference regarding angulation [10–12]; nevertheless, it should be kept in mind that all three of these studies that compared direct and indirect bonding techniques did not include radiological support for practitioners during bracket positioning.

Panoramic radiographs have been used traditionally to assess root positioning during bracket placement. However, studies have shown that erroneous root visualization could occur, due to many factors, such as distortion from non-orthogonal X-ray beams, variable facial typology between patients or even different inclinations of the patient’s head while taking the radiograph [3]. An alternative imagery is the CBCT, which allows for a three-dimensional visualization of the roots. Due to the fact that it is a fairly irradiant technique, caution must be exerted when prescribing this radiograph [3]. In this study, an ethically justified CBCT was superimposed to the patient’s maxillary 3D model, gaining visual access to the roots during the indirect bracket placement phase. The CBCT-guided indirect positioning was compared to the panoramic-guided direct positioning, and differences in angulation were observed on the laterals, which corroborates the findings by Owens et al. that the panoramic radiograph may not faithfully reproduce the actual apical position [13]. Furthermore, Andrews and Aguirre reported that orthodontists had more trouble finding correct angulation than height while positioning brackets [11, 18]. The latter also found that most positioning errors took place in the labial segment [11].

Our study showed that a significant difference was noted between conventional and self-ligating brackets, the latter generally having a higher margin of difference for each parameter. Knowing that the cost of self-ligating brackets is higher than that of conventional twin brackets, one should keep in mind the costs associated with rebonding these pre-adjusted appliances in order to obtain a correct tooth positioning [24].

This finding is also reported by Birdsall et al., who found that self-ligating brackets were more difficult to position accurately than conventional twin brackets [14].

The mini-master brackets used in this study are larger mesio-distally than the Q2 brackets. Damon brackets are designed to have larger interbracket distance than conventional twin brackets in order to promote lower forces to the teeth [25]. Andrews pointed out that the smaller the size of the bracket, the more complicated its positioning would be [18]. This may explain the difference found in this study.

It should also be noted that the level of experience of practitioners, as well as their familiarity with this type of appliance, may have influenced the present results.

In the inclusion criteria for choosing the participating orthodontists, a minimum of 2 years of clinical experience was required: This led to a discrepancy in experience levels among different participants, as some only had two years of experience, whereas others were more qualified. This discrepancy also existed for the virtual bonding, even though the participants were all calibrated by the same technician, who would also answer any technical question they had during the procedure, without influencing the results. The large number of statistical measures in this study made it difficult to allocate orthodontists according to their experience levels, notwithstanding the fact that it would have diminished any associated bias.

Regarding direct bonding, our results are concurrent with those reported by Fowler et al., who had found that the level of experience affected angulation results, but are not in agreement with those reported by Armstrong et al., who reported that it affected the height parameter only [26, 27].

Regarding virtual bracket bonding, a study by De Oliveira et al. found that angulation was affected by the level of experience of the orthodontist, which also may explain our results regarding the angular dimension [28].

In the conclusion of their study, Koo et al. stressed the importance of finding different ways of measurement than those followed in their methodology [10]. The main difference between the present study and the previous ones is the incorporation of technology; the indirect positioning was done virtually, with a 3D visual access to the roots, which facilitated brackets’ placement at the correct angulation. Study models were scanned by a digital scanner, unlike previous studies which relied on photography, and subsequently risked errors of magnification and distortion. Furthermore, the digital software used allowed us to perform a precise superimposition of the models as well as a reproductible study of the different measurements between the two techniques.

Twenty practitioners placed the brackets on 10 different teeth, with 3 measurements taken per tooth, which makes a total of 1200 measurements carried out in our study.

Given this large number of measurements, our protocol was limited to the maxillary arch. It would be interesting to include the lower arch in an ulterior study.

Although the mannequin and models were designed in order to simulate a clinical environment, it should be kept in mind that different results may be obtained on a real patient. The difficulty of finding a sufficient number of patients with malocclusions of similar difficulty, relatively healthy teeth with an ethically justified CBCT, was a factor that made this an in vitro study. A clinical trial that involves practitioners with an equal level of experience in different types of brackets would be the golden standard.

The study was conducted on brackets from right second premolar to left second premolar. Out of the three studies in the literature that compared direct and indirect bracket positioning, two studies did not include the molars: A randomized clinical trial by Hodge et al. evaluated only the six anterior teeth because error in bracket placement on these teeth would have the most serious aesthetic consequences [12]. Koo et al. conducted a study that was limited from 15 to 25 [10]. Furthermore, we did not find molar tubes that were available in both the Lebanese market and the OrthoAnalyzer™ library. It would be interesting to include the molars in an ulterior study, since a major advantage of the indirect bonding technique is easier posterior positioning [8].

One may argue that most errors in indirect bonding occur during the transfer process. However, technological advances have increased the accuracy of said transfers. Studies have shown that using specific trays can be a very precise method of bracket transfer in the digital indirect bonding technique [29–31]. Therefore, we did not include the transfer process in our methodology given that the differences between the virtual 3D setup and the result after transfer would be minimal.

Since, for the majority of teeth, the differences found between the two positioning techniques were not statistically significant, one may wonder if a switch from the direct bonding system to the three-dimensional CBCT indirect world is worth it, knowing that it is a fairly irradiant technique. Nevertheless, this technique could be of interest in individualized orthodontics: The practitioner is often faced with contralateral teeth having different initial torque values. In the straight wire technique, in order to obtain a symmetrical result, he would have to either incorporate bends in the archwire, use torquing auxiliaries, or use a hybrid brackets setup [32]. As shown in this study, technology makes it possible to achieve virtual bracket positioning with a 3D acquisition of the roots by means of a CBCT. This superposition can then be followed by customization of the bracket base or of the actual bracket slot in order to obtain an adequate inclination or torque. This is only possible by means of an indirect, individualized, digitized bonding, which underlines the importance and the advantages of this technique [33].

## Conclusion

Based on the results of the study, it appears that there are no major statistically significant differences between direct and digital indirect bonding. The difference was significant only on the laterals in terms of angulation, and on teeth 13 and 15 in terms of mesio-distal centering. It seems that bracket type does influence positioning accuracy, since self-ligating brackets had a larger error range than conventional twin brackets.

## Data Availability

The datasets used and/or analyzed during the current study are available from the corresponding author on reasonable request.

## Abbreviations

CBCT: Cone-beam computed tomography
MBT: McLaughlin Bennet Trevisi prescription
MARPE: Mini-screw Assisted Rapid Palatal Expansion
ABO: American Board of Orthodontics
